# Assessment of glycemia in chronic kidney disease

**DOI:** 10.1186/s12916-022-02316-1

**Published:** 2022-04-13

**Authors:** Mohamed Hassanein, Tariq Shafi

**Affiliations:** 1 Division of Nephrology, Department of Medicine, University of Mississippi Medical Center, 2500 North State Street, Jackson, MS 39216 USA; 2 Department of Population Health, John D. Bower School of Population Health, University of Mississippi Medical Center, 2500 North State Street, Jackson, MS 39216 USA; 3 Department of Physiology and Biophysics, University of Mississippi Medical Center, 2500 North State Street, Jackson, MS 39216 USA

**Keywords:** Glycemic monitoring, glycemic assessment, Diabetes mellitus, Chronic kidney disease, Hemoglobin A1C, Fructosamine, Glycated albumin, One, five-anhydroglucitol, Self-monitored blood glucose, Continuous glucose monitoring

## Abstract

Reliable assessment of glycemia is central to the management of diabetes. The kidneys play a vital role in maintaining glucose homeostasis through glucose filtration, reabsorption, consumption, and generation. This review article highlights the role of the kidneys in glucose metabolism and discusses the benefits, pitfalls, and evidence behind the glycemic markers in patients with chronic kidney disease. We specifically highlight the role of continuous glucose monitoring as an emerging minimally invasive technique for glycemic assessment.

## Background

Quantifying the severity and patterns of hyperglycemia are central to the diagnosis and management of diabetes [1]. Early diagnosis and treatment of prediabetes can prevent progression to diabetes, and early diagnosis and management of diabetes can prevent its long-term microvascular and macrovascular complications [2, 3]. Glycemic control is critical to detect immediate complications of glucose-lowering medications (hypoglycemia), short-term diabetes complications (diabetic ketoacidosis and hyperglycemic hyperosmolar state), and intermediate-term diabetes complications (infections) [2, 3]. Glycemic monitoring also provides insights into individual glucose patterns allowing individualized patient management [1, 4]. In this review, focused on patients with chronic kidney disease (CKD), we will discuss the unique aspects of glycemic disarray in kidney disease and summarize the evidence for different methods of assessing glycemia by indirect markers of average glycemia and glucose monitoring.

## Main text

### The role of the kidneys in glucose homeostasis

The kidneys play a vital role in maintaining glucose homeostasis through glucose filtration, reabsorption, consumption, and generation [5]. Glucose is freely filtered at the glomerulus, and in a healthy individual, all of the filtered glucose is reabsorbed [6]. Glucose reabsorption from the tubular filtrate occurs by secondary active transport by sodium-glucose cotransporters (SGLT) with 90% of the filtered glucose absorbed by SGLT2 in the S1 segment of the proximal tubule and 10% reabsorbed by SGLT1 in the latter parts of the proximal tubule [7]. The maximum rate of tubular absorption of glucose is 375 mg/min, threefold higher than the glucose filtration rate of about 125 mg/min [7]. However, this threshold varies between the nephrons, and glucose appears in the urine as the plasma level rises above 200 mg/dL [5]. In kidney disease, a low glomerular filtration rate (GFR) reduces the filtered glucose load. In contrast, the loss of functioning nephrons reduces the capacity for reabsorption. The net effect on the kidneys’ glucose threshold can be quite variable in people with kidney disease. In addition to the excretion of glucose, the kidneys also consume ~ 10% of the plasma glucose as an energy source for its metabolic functions [8]. The kidneys also contribute to about 20–25% of the circulating blood glucose in the fasting state by gluconeogenesis [5, 9–12]. The kidneys are also responsible for metabolizing insulin and clearing many glucose-lowering medications [8, 13–15]. The net effect of this interplay between kidneys, glucose metabolism, and medication clearance can be highly variable in individual patients with a propensity for both hyperglycemia due to reduced excretion and reduced consumption and fasting hypoglycemia due to reduced capacity for gluconeogenesis and prolonged half-life of glucose-lowering medications [5, 9–12, 16].

### Indirect markers of average glycemia

The indirect markers of glycemia provide an assessment of the average glucose levels over the preceding three months (hemoglobin A1C), 2 weeks (fructosamine and glycated albumin), and 1–2 weeks (1,5 anhydroglucitol) [17]. In the following sections, we discuss the biological rationale for using each marker and special considerations in patients with CKD, which might affect the markers’ diagnostic validity (association with average glucose levels) and predictive validity (association with long-term complications of diabetes).

#### Hemoglobin A1C

##### Biology

Hemoglobin A1C (A1C) is formed by the non-enzymatic attachment of glucose to the N-terminal valine of the β-chain of hemoglobin over the red blood cells’ lifespan. In healthy individuals, the average lifespan of red cells is 90 days, and therefore, A1C levels generally reflect the average blood glucose in the preceding 3 months [17]. However, A1C values are influenced more by recent glucose levels than more distant ones as the mean level of blood glucose in the 30 days preceding the A1C contributes to ~ 50% of the A1C level [18]. A1C measurements and reporting are highly standardized, and the National Glycohemoglobin Standardization Program (NGSP) ensures standardization and calibration of A1C assays, minimizing analytic variability between individual laboratories [19].

##### Special considerations in CKD

The interpretation of A1C levels in patients with CKD comes with numerous caveats (Table 1). Anemia is common in advanced CKD for multiple reasons, including reduced erythropoietin production by the kidney, inflammation, and the effect of circulating uremic toxins [21]. When anemia is treated with erythropoiesis-stimulating agents, there is an increase in circulating young red cells. Due to the shortened red cell lifespan [22] and the higher number of immature red cells, there is a shorter time for glycation, resulting in A1C being lower than expected [23, 24]. Variable effects on A1C occur due to other factors in patients with CKD, including sickle cell trait [25] (more common in Blacks [26, 27]), oxidative stress, inflammation, and acidosis [28]. A1C levels also vary according to ethnicity and race, with some evidence of higher A1C levels in non-Whites than Whites [29]. These factors influence both the diagnostic accuracy of A1C and its predictive validity in CKD.

**Table 1:** Settings in which the interpretation of AIC is problematic

Effect on A1C	Conditions
**Falsely low**	Chronic blood loss; autoimmune hemolytic anemia; thrombotic microangiopathy; **malignant hypertension**; sickle cell anemia; thalassemia; certain hemoglobinopathies; glucose-6-phosphate dehydrogenase deficiency; intra-red cell alkalosis; hypertriglyceridemia; malaria; **HIV**; other chronic infections; **hemodialysis**
**Falsely high**	**Iron deficiency**; vitamin B12 deficiency; persistence of fetal hemoglobin; **intra-red cell acidosis**; splenectomy; hyperbilirubinemia; **alcoholism**; **smoking**
**Variable effects**	**Chronic kidney disease**; pregnancy; certain hemoglobin variants; methemoglobin; **recent blood transfusion**; cancer; **chronic liver disease**
**Interfering medications**: **iron-replacement therapy**; treatment for vitamin B_12_ deficiency; anti-malarial drugs; **sulfonamides**; **aspirin**; vitamins C or E; **antiretrovirals**; ribavirin; dapsone; hydroxyurea; **chronic opioid use**	

Adapted from Diabetes in America, 3rd Edition, Table 1.5 [20]

Conditions in **bold font **represent CKD or conditions that are more common in CKD

*Abbreviations*: *A1C* hemoglobin A1C, *HIV* human immunodeficiency virus, *CKD* chronic kidney disease

##### Diagnostic accuracy

In patients with diabetes without CKD, mean glucose from multiple measurements, such as by continuous glucose monitoring (CGM), is highly correlated with A1C (rho 0.8 to 0.9) [30, 31]. Limited data are available for A1C reliability in patients with CKD. In a single-center study of 104 patients with diabetes and CKD, Zelnick et al. obtained CGM over two 6-day periods separated by 2 weeks [32]. The correlation between mean glucose and A1C was 0.85 for patients with eGFR 45–59 ml/min/1.73 m^2^ (*n* = 28), 0.91 for patients with eGFR 30–44 ml/min/1.73 m^2^ (*n* = 30), and 0.61 for patients with eGFR < 30 ml/min/1.73 m^2^ (*n* = 28). There are no CKD studies comparing A1C to glucose measured using the recommended 14-day CGM.

##### Predictive validity

There are two crucial considerations while interpreting the predictive validity of glycemic markers. First, the association of an observed *lower* marker with outcomes in observational studies is not the same as *lowering* the marker with treatment in the setting of a randomized clinical trial due to potential side effects of treatment (hypoglycemia, drug-specific toxicity). Second, with regard to observational studies, it is also important to distinguish between electronic health records (EHR)-based retrospective cohorts with clinically measured A1C, which could be confounded by indication, versus prospective cohort studies with measurement of A1C in all participants as part of the research protocol.

A1C targets in patients with CKD are based on general population observational studies and clinical trials that either did not include patients with CKD or had few patients with advanced CKD. In two retrospective EHR-based cohorts, A1C levels < 6–7% or > 9% were associated with an increased risk of death [33, 34]. In the Action to Control Cardiovascular Risk in Diabetes (ACCORD) trial, patients with CKD stages 1 to 3 (*n* = 3636) had a 1.6-to-3-fold higher risk of cardiovascular events than patients without CKD (*n* = 6506). However, in patients with CKD, intensive glucose control (achieved A1C, 6.7%) compared to standard control (achieved A1C, 7.5%) was associated with a higher risk of any cause of death and cardiovascular death but lower risk of non-fatal myocardial infarction. The risk of hypoglycemia was 2-fold higher in those with CKD than in those without CKD [35].

#### Glycated proteins

##### Biology

Similar to the non-enzymatic glycation of hemoglobin, glucose also attaches to circulating plasma proteins forming ketoamines [25]. Fructosamine represents all glycated plasma proteins. Glycated albumin is formed by the non-enzymatic attachment of glucose to the lysine residues on plasma albumin, the most abundant plasma protein. As the half-life of albumin is ~ 14 days, fructosamine and glycated albumin values represent the average glucose levels over the preceding 2–4 weeks [17, 25]. The suggested reference range for fructosamine is 205 to 285 μmol/L and is 11.9% to 15.8% for glycated albumin [36]. These values can vary between different labs due to the lack of standardized assays [37].

##### Special considerations in CKD

Both glycated albumin and fructosamine offer the advantage of not being affected by hematological factors that alter A1C levels [16, 38, 39]. However, they can be affected by protein turnover, such as in patients with significant proteinuria, hypoalbuminemia, and cirrhosis [40]. Serum albumin is also a negative acute phase reactant with acute illness leading to lower serum albumin levels after a lag of about 1–2 weeks [41]. The impact of these changes on the interpretation of the glycated protein levels has not been studied.

##### Diagnostic accuracy

In patients with diabetes without CKD, mean glucose from CGM is highly correlated with fructosamine (rho 0.85) and glycated albumin (rho 0.87), supporting their role as alternative glycemic markers [31]. The recommended 14-day CGM has not been used to evaluate the diagnostic accuracy of these markers in patients with CKD and the published CKD studies had a small sample size, limiting interpretation. The correlation reported with mean glucose was also highly variable for fructosamine (rho range 0.54 to 0.80) and glycated albumin (rho range 0.41 to 0.88) [42, 43]. In the previously noted study by Zelnick et al. [32], the correlation with mean glucose from CGM in patients with eGFR < 30 ml/min/1.73 m^2^ was 0.60 for fructosamine and 0.77 for glycated albumin, similar to the correlation with A1C (0.61).

##### Predictive validity

Non-CKD observational studies suggest that the association of fructosamine and glycated albumin with outcomes is similar to observed with A1C [43–45]. There are no studies reporting associations of glycated proteins with outcomes in patients with non-dialysis dependent CKD. Further, there are no randomized controlled trials that used glycated protein targets for glycemic control [44].

#### One, five-anhydroglucitol (1,5-AG)

##### Biology

One, five-anhydroglucitol (1,5-AG) is structurally identical to glucose with the absence of the C-1 hydroxyl group [46]. It is widely available in food sources, including soybeans, rice, pasta, fish, fruits, vegetables, tea, milk, and cheese [47]. One, five-anhydroglucitol is freely filtered at the glomerulus, and in healthy individuals, 99.9% is reabsorbed by the sodium-glucose cotransporter 4 (SGLT4) in the proximal tubules. Glucose competes with the reabsorption of 1,5-AG by SGLT4, and as hyperglycemia exceeds the kidneys’ absorption threshold, 1,5-AG reabsorption decreases, and urinary excretion increases. As a result, 1,5-AG plasma levels *fall* with hyperglycemic excursions, and low levels of 1,5-AG indicate recent (1–2 weeks) glycemic excursions. Thus, 1,5-AG levels are more reflective of plasma glucose *surges* beyond the kidneys’ reabsorption threshold during the preceding 1–2 weeks, rather than average glycemia during this period. One, five-anhydroglucitol is not useful in patients with normoglycemia and those with blood glucose levels below the threshold for glucosuria [46, 48–50]. The healthy reference range of 1,5-AG is 8.4 to 28.7 μg/mL [36].

##### Special considerations in CKD

One, five-anhydroglucitol is highly dependent on the kidneys’ threshold for glucose reabsorption. In CKD, the kidneys’ glucose threshold can be quite variable depending on the balance between filtered glucose load, determined by GFR, and the reabsorptive capacity of the remaining functioning nephrons, determined by the proximal tubular function [7]. The lack of reliability of estimated GFR versus measured GFR and the lack of methods to quantify tubular dysfunction are limitations to the using a marker of glycemia in CKD that depends on these factors. One, five-anhydroglucitol levels can also be affected by diet, which should be taken into account while interpreting the results [51].

##### Diagnostic accuracy

As 1,5-AG is a marker of postprandial hyperglycemia and glycemic surges, its diagnostic accuracy should be based on CGM used to define glucose patterns. In patients without CKD, 1,5-AG correlates with the area under the curve for glucose above 180 mg/dL (AUC-180) [49, 52, 53]. There are no studies of the diagnostic accuracy of 1,5-AG in patients with CKD.

##### Predictive validity

In the general population, low 1,5-AG (indicating glycemic surges) is associated with diabetic microvascular and macrovascular complications [54–56]. The predictive validity of 1,5-AG in patients with CKD is unknown.

#### Direct measurement of glucose

Glucose variability may contribute to the pathogenesis of the vascular complication of diabetes [57]. Glucose monitoring is required for routine clinical care of patients with diabetes prone to hypoglycemia. Glucose can be measured in whole blood, serum, plasma, capillary blood, or interstitial fluid. Plasma glucose is recommended for the diagnosis of diabetes as there are differences in glucose levels depending on the sample types [18]. The plasma glucose is 11% higher than whole blood glucose in patients with normal hematocrit due to the higher water content of plasma. However, in heparinized plasma, glucose is 5% lower than serum due to the shift of water from red cells to plasma. Capillary blood glucose levels are similar to venous blood glucose during the fasting state but can be 20–25% higher than venous blood glucose during the postprandial state. Interstitial fluid glucose levels lag ~ 10 min behind the blood glucose levels which can be important when the blood glucose levels are falling rapidly [58]. In clinical practice, direct glucose measurement by either self-measured blood glucose (SMBG) or CGM provides information on acute glucose excursions and glycemic variability.

#### Self-measured blood glucose (SMBG)

##### Technique and diagnostic accuracy

Glucose meters for SMBG are widely available, but not all Food and Drug Administration (FDA)-cleared glucose monitors have similar reliability. Home-use devices can differ from professional use devices, and while most quantify plasma glucose, some may quantify whole blood glucose [1]. Several factors affect the diagnostic accuracy of SMBG, including glucose strips, physical, patient, and pharmacological factors [59]. SMBG uses electrochemical glucose oxidase strips, which are sensitive to oxygen concentration. When glucose interacts with glucose oxidase, a series of electrochemical reactions lead to glucose signaling and a glucose reading. Strip to strip variation and changes in glucose oxidase enzyme coverage can lead to inaccuracies in glucose measurement. Altitude and temperature alter oxygen concentration and capillary circulation, respectively, leading to inaccuracies in glucose readings. Patient-related factors such as the ability of patients to use the correct technique and differences in hematocrit values can also lead to errors in glucose readings. Red blood cells harbor significant amounts of glucose, leading to spurious readings if glucose meters do not correct for it. High triglycerides take up volume, reducing glucose in the capillary volume, while uric acid can be oxidized by glucose oxidase, leading to falsely elevated blood glucose values. Medications such as acetaminophen, ascorbic acid, L-dopa, and Tolazamide can interact with the electrodes on glucose strips altering glucose readings [59].

##### Special considerations in CKD

Hyperuricemia and gout are common in CKD, and elevated uric acid is oxidized by the glucose oxidase monitor electrode and counted as glucose leading to falsely high blood glucose readings [59–61]. Severe hypoglycemic encephalopathy in a patient with CKD, masked by hyperuricemia, has been described [62]. Pain is common in patients with neuropathy and osteoarthritis and is preferentially treated with acetaminophen in patients with CKD [63]. Glucose dehydrogenase monitors are not affected by uric acid and acetaminophen but provide falsely high glucose readings in patients treated with icodextrin containing peritoneal dialysis solutions [64].

#### Continuous glucose monitoring

##### Technique

CGM is a minimally invasive modality for glycemic monitoring. There are two types of CGMs, real-time CGM (RT-CGM) and intermittently scanning CGM (IS-CGM). Real-time CGM automatically transmits continuous CGM readings to the user providing real-time values that are sent to a receiver or smart device. Intermittently scanning CGM scans for glucose readings only when the user prompts the device to scan [65]. Many CGM wearable sensors are now factory-calibrated, eliminating the need for fingersticks [66, 67]. These sensors are approximately the size of a quarter, weigh < 5 grams, and have a < 0.4 mm thin filament inserted into the skin surface to measure interstitial fluid glucose. The sensors are water-resistant; the person wearing them can shower and swim. CGM sensors measure interstitial glucose levels several times per hour and are being widely used in clinical practice and research [68–70]. CGM devices include personal and professional CGM devices. Personal CGM devices typically record and transmit glucose values to a personal receiver which alerts the patient. Personal CGM devices can also be programmed to share information with caregivers, clinicians, and family members. Professional CGM devices are wearable CGM devices that are provided to patients to analyze and record glucose values, typically over a 2-week period. These devices can provide valuable information for titrating glucose-lowering medications, particularly those that predispose to hypoglycemia [71].

##### CGM interpretation

The metrics for reporting and interpretation of CGM were recently standardized [72]. The key elements in the interpretation of CGM include time in range, time above target, and time below target. Time in range is the percentage and time of glucose readings within the target range (70–180 mg/dL). Time above range is the percentage and time of readings above target glucose range (level 1: 181–250 mg/dL, level 2: > 250 mg/dL). Time below target is the percentage and time of readings below the target glucose range (level 1: 54–69 mg/dL, level 2: < 54 mg/dL). The main goal of using CGM for glycemic monitoring is to maximize the TIR while minimizing time above or time below target [72]. The glucose values from CGM are also converted to an estimated A1C, referred to as the glucose management indicator (GMI), to distinguish it from laboratory-measured A1C. The results are provided as a single-page ambulatory glucose profile [1].

##### Special considerations in patients with CKD

**Fig. 1:**
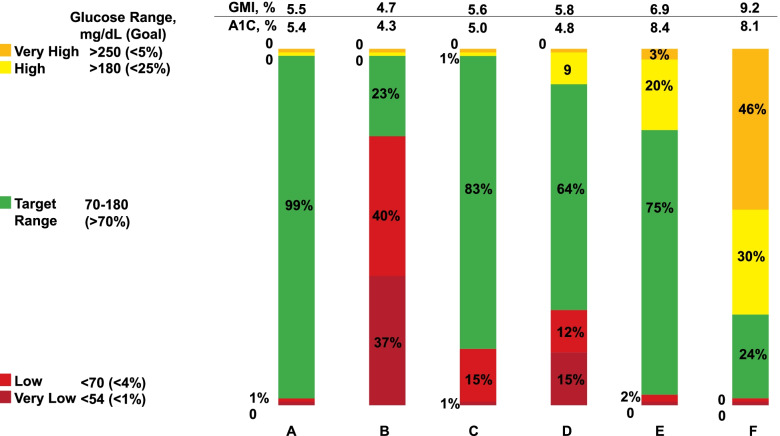
Time in range results from continuous glucose monitoring in patients with chronic kidney disease. Continuous glucose monitoring (CGM) data from 6 individuals, including one person without diabetes (**A**) and five persons with diabetes and end-stage kidney disease (**B**–**F**), are presented. The CGM data are categorized as time-in-range, based on consensus recommendations. Glucose management indicator (GMI) is calculated from the average glucose results for each person, and lab-measured A1C is also presented. Note that significant variability in the glucose time-in-range is present within the same range of A1Cs (**A**–**D** and **E**, **F**). For example, for person **E** with an A1C of 8.4%, the GMI is 6.9%, and the CGM time-in-range is within acceptable limits. However, for person **F**, the A1C is 8.1%, GMI is 9.2%, and the CGM time-in-range is unacceptably high

**Fig. 2:**
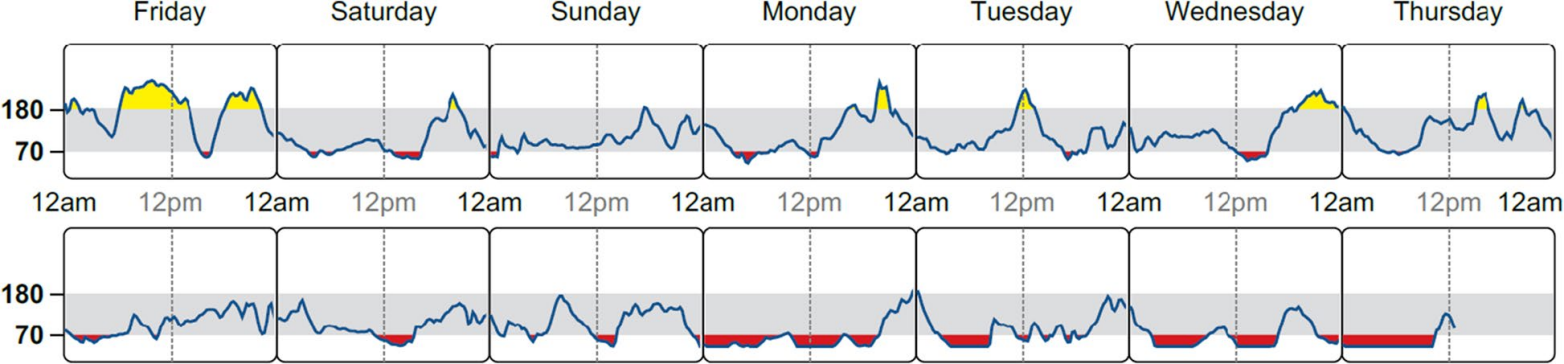
Daily glucose profile from continuous glucose monitoring. Continuous glucose monitoring profile for a person with diabetes and end-stage kidney disease. The time-in-range results are shown in Fig. 1D. The profile shows that the hyperglycemic excursions during week 1 (top panel, yellow color indicating glucose above 180 mg/dL) decreased, but there is a trend towards more nocturnal hypoglycemia during week 2 (bottom panel, red color indicating glucose below 70 mg/dL), particularly during the last 4 days of the monitoring period. Patient care can be individualized using this information, combined with dietary history and glucose-lowering medications use

Glucose variability may be higher in patients with CKD. Postprandial hyperglycemia can occur due to lack of kidneys’ filtration and clearance of glucose. Fasting hypoglycemia can occur due to the lack of kidney gluconeogenesis and prolonged half-life of endogenous insulin and glucose-lowering medications. Biomarkers of average glycemia, including A1C and glycated proteins, might also not be reliable in advanced CKD due to limitations discussed previously. CGM can provide valuable insights into glucose patterns and glycemic control that might not otherwise be available. The discrepancy between GMI and laboratory-measured A1C can also be valuable in making treatment decisions, including intensifying and de-escalation of glucose-lowering medications. Figure 1 shows the CGM profile of a person without diabetes (Fig. 1A) and five patients with end-stage kidney disease (Fig. 1B–F). The glucose time-in-range provides invaluable information, not fully captured by either A1C or GMI. Detailed daily glucose data, a food diary, and glucose-lowering medication dosage provide further information to guide management (Fig. 2). Rigorous studies of patients with advanced CKD are needed to determine if CGM should replace the measurement of plasma biomarkers to assess glycemic control.

##### Glycemic assessment: KDIGO guidelines

The Kidney Diseases Improving Global Outcomes (KDIGO) group recently updated the guidelines for the management of diabetes in CKD. The guidelines recommend the use of A1C to assess glycemic control in patients with CKD. The recommendation is graded Level 1 (We Recommend) C (Low Quality of Evidence). The recommendation is based on the totality of evidence supporting the use of A1C in the general population, including A1C’s diagnostic and predictive validity and A1C’s use as a treatment target in clinical trials. The guidelines note the unreliability of A1C in patients with advanced CKD (eGFR < 30 ml/min/1.73 m^2^ and kidney failure treated with dialysis). Alternative biomarkers of glycemia are not recommended due to the lack of prospective observational or clinical trial data in patients with CKD [28].

The KDIGO guidelines also support the use of CGM in patients with CKD. They suggest using CGM to calculate GMI in patients with advanced CKD. CGM can also be considered in earlier stages of CKD if there is a clinical concern that the A1C is not reflective of the patient’s glycemic control, such as discordance between A1C and SMBG or random glucose, or symptoms of hypoglycemia or hyperglycemia. CGM-derived GMI can be compared to A1C, with the caveat that the relationship between the two may differ over time [28]. Table 2 depicts the advantages and disadvantages of different modalities for glycemic assessment.

**Table 2:** Advantages and disadvantages of different methods for glycemic assessment in patients with chronic kidney disease

Modality	Advantages	Disadvantages
**Hemoglobin A1C**	- Widely available - Standardized assays - Reflects glycemic status over the previous 90 days - Clinical trial outcomes data - Limitations are well known	- Unreliable in many comorbidities associated with CKD, including anemia - Unreliable in advanced CKD (eGFR < 30 ml/min/1.73 m^2^)
**Glycated proteins (fructosamine, glycated albumin)**	- Assays are commercially available - Reflect glycemic control over the previous 2–4 weeks - Useful in the presence of anemia or use of erythropoietin supplementation agents	- Assays are not standardized - Protein turnover may limit use in patients with proteinuric CKD - Clinical trial outcome data are limited
**1,5 AG**	- Reflects glycemic control over the previous 2 weeks - Useful in detecting glycemic excursions	- Assays are not standardized - Lack of data on accuracy and diagnostic validity - Clinical trial outcome data are limited
**SMBG**	- Widely available and FDA approved. - Reliable measurement of current glucose level - Clinical trial outcome data	- Diagnostic accuracy is affected by strip-to-strip variation, altitude, temperature, hyperuricemia, and some medications - The discomfort from fingersticks limits use
**CGM**	- Provides detailed snapshot of glycemic control over the duration of sensor - Allows care to be individualized to each patient - Clinical trials outcomes data are emerging	- Cost is higher compared to other tests - Lack of familiarity limits use by non-endocrinologists

*Abbreviations*: *CKD* chronic kidney disease, *eGFR* estimated glomerular filtration rate, *A1C* hemoglobin A1C, *1,5AG* one, five-anhydroglucitol, *SMBG* self-monitored blood glucose, *CGM* continuous glucose monitoring

## Conclusions

Glycemic monitoring is essential to prevent complications and improve outcomes in patients with diabetes. A1C has been the gold standard in monitoring blood glucose levels in patients with CKD, but it may be inaccurate in multiple comorbidities present in patients with CKD. Fructosamine, glycated albumin, and 1,5-AG have been proposed as alternative markers in patients with diabetes. However, these markers have not been rigorously studied in patients with CKD. CGM is available as a promising minimally invasive technique that avoids the pitfalls of routing fingerstick glucose monitoring and assesses blood glucose levels continuously. Prospective studies are warranted to validate CGM’s efficacy in patients with CKD.

## Data Availability

Not applicable.
